# Evaluation of Different Antiretroviral Drug Protocols on Naturally Infected Feline Immunodeficiency Virus (FIV) Cats in the late Phase of the Asymptomatic Stage of Infection

**DOI:** 10.3390/v4060924

**Published:** 2012-05-30

**Authors:** Nélida V. Gómez, Adriana Fontanals, Víctor Castillo, María A. Gisbert, Adriana Suraniti, Graciela Mira, Paola B. Pisano

**Affiliations:** 1 Service of Electrophysiology, Veterinary Medicine Teaching Hospital of the University of Buenos Aires, San Martín Av. 4453, CP 1427, Buenos Aires, Argentina; Email: asuraniti@med.fvet.uba.ar; 2 Department of Small Animals Medicine, School of Veterinary Sciences, University of Buenos Aires, CP 1427, Buenos Aires, Argentina; Email: clinpeq@fvet.uba.ar (V.C.); gisbertma@hotmail.com (M.A.G.); paolabpisano@hotmail.com (P.B.P.); 3 Department of Clinical Pathology. School of Veterinary Sciences, University of Buenos Aires, Buenos Aires, 1427, Argentina; Email: gmira@fvet.uba.ar; 4 Department of Immunology, School of Veterinary Sciences, University of Buenos Aires, CP 1427, Buenos Aires, Argentina; Email: afontanals@fvet.uba.ar; 5 Pablo Cassará Laboratory, Saladillo 2452, Buenos Aires, Argentina

**Keywords:** antiretroviral therapy, FIV, retrovirus, visual and auditory evoked potentials, ZDV, 3TC, valproic acid, rHuIFN-α

## Abstract

The aim of this study was to evaluate the efficacy of the antiretrovirals: Zidovudine (ZDV) alone; ZDV + Recombinant Human Interferon*-*α (rHuIFN-α); ZDV + Lamivudine (3TC) and ZDV + valproic acid (Valp) on naturally feline immunodeficiency virus (FIV)-infected cats, in the late phase of the asymptomatic stage of infection. The follow-up was performed over one year, through clinical evaluation and the determination of viral loads and CD4+/CD8+ ratios. Neurological signs were studied by visual and auditory evoked potentials (VEP, AEP) and the responses were abnormal in 80% of the FIV-infected cats. After one year, an improvement in VEP and AEP was observed in the ZDV + Valp group and a worsening in the group receiving ZDV + rHuIFN-α. The CD4+/CD8+ ratio showed a significant increase (both intra and inter-groups) only in ZDV and ZDV + 3TC, between their pre-treatment and one year values, as well as among the other groups. Viral load only showed a significant decrease in ZDV and ZDV + 3TC groups, when comparing the values at one year of treatment *vs.* pre-treatment values and when the different groups were compared. In addition, the viral load decrease was significantly more pronounced in the ZDV + 3TC *vs.* ZDV group. We conclude that ZDV and ZDV + 3TC produce significant reductions in viral load and stimulate a recovery of the CD4+/CD8+ ratio, compared with the other protocols. It is clear that the addition of 3TC resulted in a greater reduction in viral load than use of ZDV as a single drug. Therefore, the combination ZDV + 3TC could be more effective than the sole use of ZDV.

## 1. Introduction

Since the appearance of the human immunodeficiency virus (HIV), cats infected with feline immunodeficiency virus (FIV) have been considered an excellent animal model for the study of the disease and many drug protocols are evaluated in cats to establish antiretroviral efficiency and low toxicity in humans. 

In HIV-infected individuals, the best results are obtained with HAART (Highly Active Antiretroviral Therapy), which combines drugs that act at critical points of the retroviral replication [1,2,3]. Drug combinations decrease the risk of the emergence of drug resistance that is commonly associated with single drug therapy [1,3]. Several reverse transcriptase inhibitors effective against HIV are also active against FIV, allowing successful use of the cat model to investigate drug interactions and resistance development [2]. 

Zidovudine (ZDV) (nucleoside analog) is the reverse transcriptase inhibitor most studied in cats. ZDV administration has resulted in decreased viral load, improved clinical condition and better quality of life, but it may induce anemia, vomiting and anorexia [1]. 

Lamivudine (3TC) is another nucleoside analog that allows for longer periods of effective treatment and combines synergistically with ZDV [1,2]. 

The similarity between the development of resistance to ZDV in FIV and HIV-1 infections makes it reasonable to consider 3TC in combined treatment for FIV-infected cats, as it is used in combined therapy in HIV infection, [1,2].

Other studies propose the inclusion of protease inhibitors, such as TL3, that may avoid neurological clinical signs and which are more efficient in the treatment of FIV infection than HIV infection [3,4]. TL3 is well-tolerated in cats, is effective in decreasing FIV viral load and can reduce the CNS alterations noted with FIV infection, as well as the viral load and secondary responses to virus infection at the periphery [4]. 

Valproic acid (Valp), an anticonvulsant drug used in humans and animals, has been shown to inhibit the enzyme histone deacetylase. Valp activates latent pro-viruses, thereby allowing other drugs to target them [5,6]. 

Non-specific immune modulators have also been proposed for the treatment of diseases associated with FIV infection, given that the virus causes a marked deterioration of the immune system. However, long-term field studies to determine the benefits and disadvantages of immune-stimulants are still lacking [6,7,8]. Interferon-α (IFN-α) has been proposed for HIV treatment as both an antiviral and an immune-stimulant. Asymptomatic, non-progressive HIV infected patients have increased IFN-α concentrations, which correlate to a higher CD4+ T-cell count, low HIV titers, and the absence of opportunistic infections [9,10,11]. It should be highlighted that currently, IFN-α is proposed for the treatment of HIV patients only when they are infected by hepatitis C virus or in cases of Kaposi's sarcoma and as a combined therapy of IFN-α with different antiretroviral drugs [9]. 

For some time, IFNs have been proposed as an alternative for the treatment of feline retrovirus infection. Two molecules of type I IFN are currently being used: human recombinant alpha interferon (rHuIFN-α) and feline recombinant omega interferon (rFeIFN-ω) [12]. Longitudinal studies on the treatment of FIV-infected cats are scarce. In the UK, a controlled study in which 5 sick FIV-infected cats were administered rHuIFN-α and followed over a 1.5-year period did not detect significant differences in viral load and CD4+/CD8+ ratio [13]. In another study, 30 FIV-infected cats with advanced clinical disease were treated with rHuIFN-α and placebo. Interferon treatment improved the general condition of the cats, increased CD4^+^ T-cell counts, and produced a slow but progressive increase of CD8^+^ T-cell counts. However, no significant differences were observed in the viral loads [14]. Although there is currently no conclusive data, it has been proposed that the beneficial effects observed when treating with rFeIFN-ω could be due to the improvement in the animal’s immune status rather than to a direct antiviral effect [15]. However, a non-specific stimulation of the immune system may be contraindicated because it could lead to a rise in viral replication produced by the activation of lymphocytes and macrophages harboring latent infections and therefore accelerate disease progression [12,16,17]. 

Ideally, treatment should be started when FIV-infected cats are about to enter the final stage of the disease [4,6,10,18]. When the patient is still in the asymptomatic stage, their immune system is still fully functional and organs are not damaged, such that therapy is better tolerated and they do not show severe complications in their general condition, nor do they have secondary diseases due to opportunistic agents, such as *Hemobartonella felis*, *Toxoplasma gondii*, *Mycobacterium bovis*, *Cryptococcus neoformans*, *etc.* [18].

FIV invades the central nervous system (CNS) and has been detected in the brain at the time of acute infection. This was demonstrated following experimental infection with FIV Petaluma. Since these observations, several other FIV strains have been reported to cause neuropathology in domestic cats, however, cats often lack clinical signs of neuropathology [19,20]. The evaluation of visual evoked potentials (VEP) and auditory evoked potentials (AEP) can be important predictors of neurological disease, since there is a high prevalence of neurological alterations, and also because neurological disease often lacks clinical signs [21]. Highly active anti-retroviral therapy (HAART) sometimes decreases the severity of CNS disease, but in others neurological disease progresses. This is due, in part, to poor penetration of anti-retroviral compounds across the blood-brain barrier [20]. TL-3 treatment has been proposed in order to prevent the onset and progression of functional CNS pathology and because the drug normalizes the delays observed in AEPs [4]. 

In this study, we report the comparative results of combinations of antiviral drugs on naturally infected FIV cats in the late phase of the asymptomatic stage of the disease (one year post-commencement of therapy). The drug combinations evaluated were: ZDV alone; ZDV + Valp; ZDV + rHuIFN-α and ZDV + 3TC.

## 2. Results and Discussion

### 2.1. Evolution of Clinical Condition, Throughout the Course of the Study

At the beginning of the study, gingivitis and lymphadenopathy were observed in 90% of cats (29/32): 6/8 cats on ZDV alone; 8/8 on ZDV + Valp; 8/8 on ZDV + rHuIFN-α; and 7/8 on ZDV + 3TC. Both conditions improved throughout the year of therapy. The improvement in gingivitis was evident because of its serious consequences to the animals, resulting in the development of pseudoanorexia. A decrease in lesion severity or complete improvement in oral-cavity lesions was observed. As a result, patients were able to eat with ease and gained weight. In a few cases, it was necessary to implement palliative and symptomatic protocols (antibiotics, anti-inflammatory drugs, and fluid therapy) for short periods of time.

At the beginning of the study, these animals were healthy, but hyperglobulinemia was detected in a high proportion of cats (94%). The percentage of cats with hyperglobulinemia did not change through the course of the study.

Throughout the course of the study, the following medical conditions were observed: uveitis in 4/32 cats (1/8 on ZDV alone, 1/8 on ZDV + 3TC, and 2/8 on ZDV + Valp); superficial pyoderma in 3/32 cats (1/8 on ZDV + rHuIFN-α, and 2/8 on ZDV + Valp); viral rhinitis and conjunctivitis in 2/32 cats (1/8 on ZDV alone, and 1/8 on ZDV + Valp); pneumonia in 1/32 cats (1/8 on ZDV + Valp); tumors (lymphoma and squamous cell carcinoma) in 2/32 cats (1/8 on ZDV + rHuIFN-α, and 1/8 on ZDV + Valp); neurological signs, such as partial seizures and/or behavioral disorders in 5/32 cats (1/8 on ZDV alone, 2/8 on ZDV + rHuIFN-α, 1/8 on ZDV + 3TC, and 1/8 on ZDV + Valp); and generalized seizures in 4/32 cats (4/8 on ZDV + rHuIFN-α). Also, non-regenerative anemia was observed in 2/32 cats (1/8 on ZDV + 3TC, and 1/8 on ZDV + Valp). This condition improved with 100 UI/kg of human recombinant erythropoietin (rHuEPO) administered 3 times a week for a period of one month. 

Opportunist infections were not detected in these patients throughout the year of therapy and all of them remained alive and most of them in good general condition.

The aforementioned clinical findings are summarized in Table 1.

**Table 1:** Clinical signs noted in the study population throughout the year of study. ZDV =Zidovudine; Valp = Valproic acid; rHuIFN-α =Recombinant Human Interferon*-*α; 3TC = Lamivudine.

Medical conditions	ZDV	ZDV + Valp	ZDV + IFN-α	ZDV + 3TC
Uveitis	1/8	2/8	--	1/8
Superficial pyoderma	--	2/8	1/8	--
Viral rhinitis and conjunctivitis	1/8	1/8	--	--
Viral pneumonia	--	1/8	--	--
Tumors (lymphoma and squamous cell carcinoma)	--	1/8	1/8	--
Partial seizures and behavior changes	1/8	1/8	2/8	1/8
Generalized seizures	--		4/8	--
Non-regenerative anemia	--	1/8	--	1/8

### 2.2. Effect of Treatment upon Viral Load and CD4+/CD8+ Ratio

**Figure 1:**
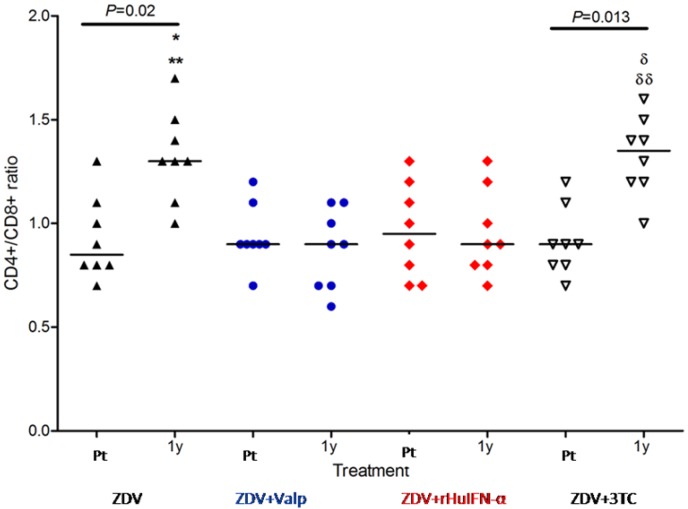
CD4+/CD8+ ratios in cats treated with either Zidovudine (ZDV) alone or in combination with Valproic acid (Valp), Recombinant Human Interferon*-*α (rHuIFN-α), or Lamivudine (3TC). CD4+/CD8+ ratios did not differ significantly between the study groups prior to treatment (Pt). Significant differences were observed at one year (1y) of treatment in comparisons between ** ZDV and ZDV + Valp (*p* < 0.01) or between * ZDV and ZDV + rHuIFN-α (*p* < 0.05). Differences were also noted between groups receiving combination therapies; ^δδ^ZDV + 3TC and ZDV + Valp (*p* < 0.01) and ^δ^ZDV + 3TC *vs.* ZDV + rHuIFN-α (*P* < 0.05). Intra-group analysis of Pt *vs.* 1y values showed significant differences between the ZDV and the ZDV + 3TC groups alone. Each dot represents one cat and the values are expressed as median and range. Symbols: apex-up black triangles: ZDV as the sole drug; blue circles: ZDV + Valp; red diamonds: ZDV + rHuIFN-α; apex-down white triangles: ZDV + 3TC.

The intra-group analysis of CD4+/CD8+ ratio (pre-treatment values *vs.* one year values) showed significant differences in ZDV group (*p* = 0.02) and ZDV + 3TC (*p* = 0.013) (Figure 1). In contrast, inter group comparisons revealed no significant differences in the pre-treatment CD4+/CD8+ ratios of the 4 groups. After one year of treatment, the groups receiving ZDV and ZDV + 3TC showed a significant increase in their CD4+/CD8+ ratios compared with pre-treatment levels. In the groups receiving Valp or rHuIFN in addition to ZDV, no increase in the CD4+/CD8+ ratio was observed after one year of treatment. (*p* < 0.05) (Figure 1). 

**Figure 2:**
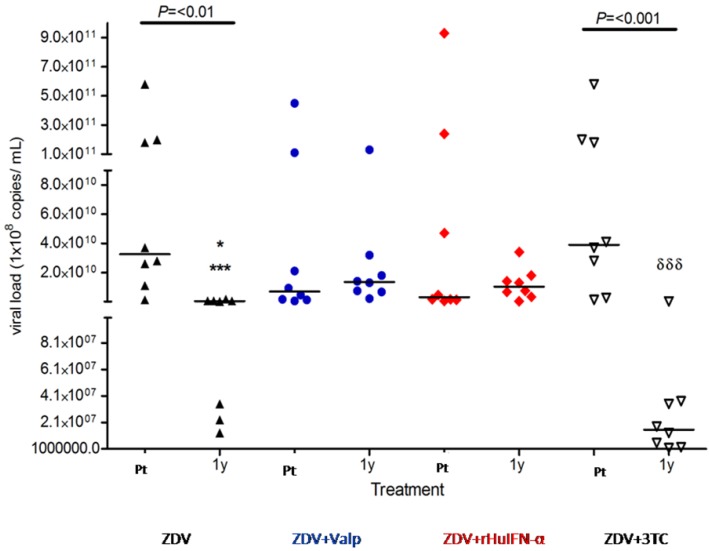
Viral load in cats treated with ZDV alone, or in combination with Valp, rHuIFN-α or 3TC. No significant differences were observed between the pre-treatment (Pt) values of each group. Significant differences were observed at one year (1y) of treatment for * ZDV *vs.* ZDV + 3TC (P = 0.03); *** ZDV *vs.* ZDV + Valp (*p* < 0.001) or ZDV *vs.* ZDV + rHuIFN-α (*p* < 0.001); ^δδδ^ZDV + 3TC *vs.* ZDV + Valp (*p* < 0.001) or ZDV + 3TC *vs.* ZDV + rHuIFN-α (*p* < 0.001). Intra-group analysis of pre-treatment *vs.* 1y values only revealed significant differences in the ZDV and ZDV + 3TC groups as indicated in the figure. Each dot represents one cat and values are expressed as median and range. Symbols: apex-up black triangles: ZDV as the sole drug; blue circles: ZDV + Valp; red diamonds: ZDV + rHuIFN-α; apex-down white triangles: ZDV + 3TC.

When viral loads were compared (Figure 2), the intra-group analysis showed a significant decrease between pre-treatment values *vs.* one year post-treatment in the ZDV (P < 0.01) and in the ZDV + 3TC (*p* < 0.001) groups. Conversely, the inter-group analysis did not reveal significant differences between the pre-treatment values of each group. 

One year post-commencement of treatment, significant differences were observed between ZDV and ZDV + Valp (*p* < 0.001), and between ZDV and either ZDV + rHuIFN-α (*p* < 0.001) or ZDV + 3TC (*p* = 0.03). Viral loads in the combined treatment group of ZDV + 3TC were significantly different from those in animals treated with ZDV + Valp (*p* < 0.001) or ZDV + rHuIFN-α (*p* < 0.001).

### 2.3. Visual Evoked Potentials (VEP) and Auditory Evoked Potentials (AEP)

**Figure 3:**
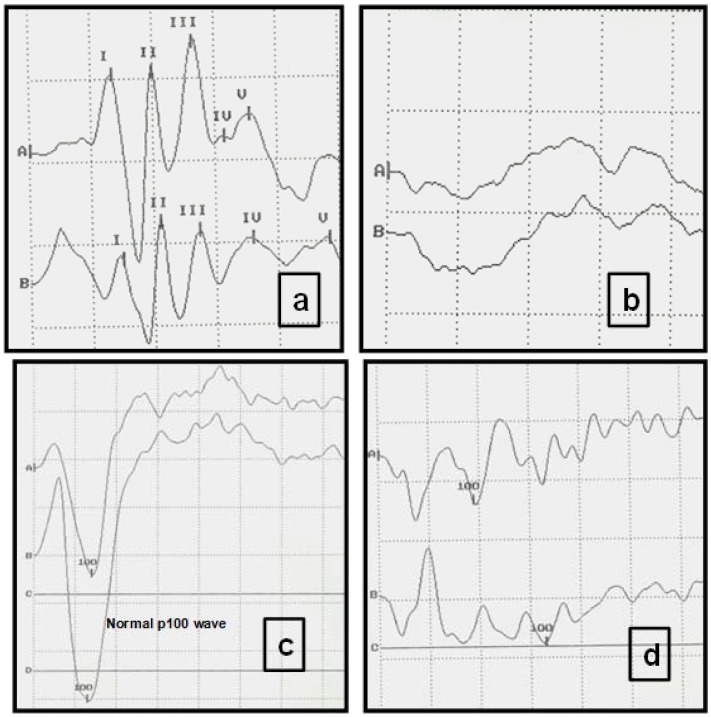
Typical visual evoked potentials (VEP) and auditory evoked potentials (AEP) of study cats displaying normal and abnormal registers. (**a**) Normal AEP in a cat. Intensity of clicks: 90 decibels (db). Each Roman numeral represents the five normal waves. Scale: Horizontal division: 1 msec; vertical division: 0.73 µV. (A right ear, B left ear). (**b**) AEP from a FIV-positive cat, with absence of recognizable recordings and abnormal neurological examination. Clicks (intensity 90 db). Each trace is an average of responses to 1000 clicks. Scale: Horizontal division: 1 msec; vertical division: 0.73 µV. (**c**) Normal VEPs in a cat. Scale: Horizontal division: 25 msec; vertical division: 5.8 µV. (A right eye, B left eye). (**d**) VEPs recorded from an FIV-positive cat. Peak amplitude (P100) is significantly decreased. Scale: Horizontal division: 25 msec. vertical division: 5.8 µV.

Pre-treatment VEP and AEP evaluations revealed abnormal evoked potentials in 80% of animals. In the majority of cases, neurological signs were either absent or evident at a low level. After one year of treatment, a modest improvement in evoked potentials was observed in the ZDV + Valp group. No changes were detected after one year of treatment in the groups receiving either ZDV alone or the combination of ZDV + 3TC. In the group receiving ZDV + rHuIFN-α, a worsening of neurological signs (for example generalized seizures), was revealed in 4/8 cats through both clinical examination and analysis of evoked potentials studies (Table 2). The most common alteration observed in the VEP of the FIV-infected cats was a reduced amplitude and a prolonged P100 wave latency. VEP showed a decreased P100 wave, with values lower than 114 msec. (normal value of latency = 140 msec.) (Figure 3b). In comparison, AEP displayed an increased time of central conduction (TCC), with values almost two-fold those of normal TCC values (up to 5 msec.), and latencies diminished by up to 30% (Figure 3, Table 2).

**Table 2:** Proportion of cats with abnormal evoked potentials in each group prior to commencement of treatment (Pt) and one year post-treatment (1y).

Evoked potentials	ZDV Pt	ZDV 1y	ZDV+Valp Pt	ZDV+Valp 1y	ZDV+IFN Pt	ZDV+IFN 1y	ZDV+3TC Pt	ZDV+3TC 1y
VEP	4/8	4/8	4/8	2/8	2/8	4/8	4/8	4/8
AEP	2/8	2/8	3/8	1/8	2/8	5/8	3/8	3/8

Upon comparing the data displayed in Table 1 and Table 2, at the beginning of the study the neurological signs were infrequent in each group (behavioral changes and partial seizures: 5/32 cats, and generalized seizures: 4/32 cats). When the evoked potentials were determined in all the patients before treatment (Table 2), 18/32 displayed altered VEPs and 10/32 displayed abnormal AEPs. These data demonstrate the importance of AEP and VEP analyses in the detection of early neurological disease in FIV-infected cats prior to clinical signs manifesting. After treatment, VEP and AEP analyses revealed a worsening in the group receiving ZDV + rHuIFN-α and an improvement in the group receiving ZDV + Valp. Neither the group receiving ZDV alone nor the group receiving ZDV + 3TC displayed alterations in evoked potentials when comparing the pre and post-treatment evaluations.

### 2.4. Side-Effects of the Therapies

At the beginning of treatment, 5 cats experienced vomiting (1/8 on ZDV alone, 2/8 on ZDV + rHuIFN-α, and 1/8 on ZDV + 3TC), 2 cats presented with anorexia (2/8 on ZDV + 3TC), and 2 cats with anemia (1/8 on ZDV + Valp, and 1/8 on ZDV + 3TC). These clinical signs were likely treatment-related side-effects. Accordingly, a rapid improvement was observed in response to therapeutic interventions and the withdrawal of treatment was not necessary.

### 2.5. Discussion

In recent years, many antiretroviral drugs have been shown to be effective in cats infected experimentally with FIV and have demonstrated a low toxicity. However there are few clinical trials that have studied the effects of antiretroviral drugs throughout the course of chronic infection, this being significant considering that long-term combination treatment would make it possible to decrease the prevalence of mutations and drug resistance [2]. Carrying out a long-term evaluation of combination antiretroviral therapy in cats presents difficulties to veterinarians because of the complex techniques required for monitoring disease progress; flow cytometry for CD4+/CD8+ ratio calculation, quantitative PCR or real-time PCR to quantify viral loads, and detailed electrophysiological analyses to measure evoked potentials. In addition, the use of combined antiretroviral drugs requires a long period of evaluation and control in the same way as is currently applied to the treatment of HIV-infected individuals. 

It is also critical that the antiretroviral therapy is administered to the cat in the later stages of the “asymptomatic” phase of infection, during which the cat may be displaying either no or limited clinical signs of disease. This is because at this stage of the disease, the immune system of the cat is relatively normal and thus may be more likely to respond to treatment. When the CD4+/CD8+ ratio falls below 0.9, the viral load increases markedly and clinical signs of immunosuppression begin to appear; cats are considered to be in the late phase of the “asymptomatic” stage and treatment must then be started.

In the present work, no significant differences were observed between the viral loads and the CD4+/CD8+ ratios of each of the study groups prior to treatment (Figure 1 and Figure 2). These findings are consistent with the cats being at a similar stage of the disease prior to commencement of treatment. 

A significant increase in the CD4+/CD8+ ratio in the ZDV group was observed, consistent with previous findings [2,3,22,23]. ZDV is well-tolerated by cats and brought about a rapid improvement in clinical condition. In the same group, viral load decreased significantly after 1 year of therapy. These benefits were of limited durability because of incomplete virus suppression and the emergence of resistant FIV strains. Although no significant difference in the CD4+/CD8+ ratio at one year post-treatment was observed between the groups receiving ZDV alone or the combination of ZDV + 3TC, viral loads showed a more significant decrease in the ZDV + 3TC-treated group. When comparing groups, it is important that both the viral load and CD4+/CD8+ ratio are compared. The CD4+/CD8+ ratio is susceptible to fluctuations unrelated to retroviral infection. The analysis of both results in combination allowed us to conclude that the combination of ZDV + 3TC was more effective across a one year treatment cycle. It was evident that the addition of 3TC greatly improved the response obtained with ZDV, consistent with previous studies [1]. Discrepancies regarding the efficacy of ZDV + 3TC as therapeutic agents may be the result of differences in the set up and conduct of respective trials. Our study suggests that commencement of therapy during the late stage of the “asymptomatic” phase of infection is critical to a successful outcome. Studies of the results of the proposed combination of drugs on other stages of the disease are still lacking. 

No significant differences were observed in the CD4+/CD8+ ratio and viral load within the ZDV + Valp group. In theory, the use of valproic acid in combination with ZDV should prove beneficial as valproic acid may activate latent proviruses, thus allowing the elimination of viral reservoirs by ZDV [6]. However, our results and similar studies in HIV infected individuals did not reveal any evidence in support of this hypothesis [22,24]. Moreover, during one year of treatment, and in comparison to treatment with ZDV alone, the survival curve showed that the ZDV + Valp group had a high mortality rate during the assessment period in contrast with the ZDV alone group [22,23].

IFN is used in a limited number of patients infected with HIV in which the disease is associated with Kaposi’s sarcoma or hepatitis C [11], however, in the current study, treatment with ZDV + rHuIFN-α affected neither the CD4+/CD8+ ratio nor the viral load. The use of IFN in HIV patients is controversial, while other studies have shown clinical and immunological benefits of anti-IFN-α immunization in HIV-infected patients. However, it has been suggested that IFN-α could produce immunosuppression rather than immune-stimulation in HIV-infected patients. Thus, any anti-viral effects that IFN-α may have in HIV infection could be eclipsed by detrimental effects to the immune system [11]. Furthermore, in addition to their therapeutic effects, IFNs commonly cause a range of side effects [11]. 

A single study has suggested that low-dose rHuIFN-α treatment may significantly prolong the survival of FIV-infected cats and may bring about a rapid improvement of the disease [14]. The improvement of clinical conditions was correlated with neither plasma viremia nor leukocyte proviral load, however a normalization of CD4+ count was observed [14]. The results of the current study found that rHuIFN-α did not ameliorate the disease. Furthermore, the effect of IFN on FIV-infected cats is not specific to retrovirus treatment, unlike antiretroviral therapy. Our observations in the ZDV + rHuIFN-α group revealed a worsening of both neurological signs and evoked potentials. These findings were in contrast to the improvements observed in the ZDV + Valp group, which reflected, presumably, the depressor effect of valproic acid on the CNS. 

Electrophysiological parameters suggested a slowing-down of nervous transmission, as revealed by changes to latencies, amplitudes and an increase in the time of central conduction. These findings could be the result of the lymphoproliferative reaction induced by virus activity in the CNS [20]. Evoked potentials were shown to be very useful in the detection of neurological signs in FIV-infected cats, and could be considered an effective means of predicting early neurological disease, which frequently lacks clinical signs [21]. The study of evoked potentials establishes neurological deterioration (or improvement) sensitively, even when clinical signs may not be evident. By comparing Table 1 and Table 2, it can be seen that at the beginning of the study, there was a low incidence of neurological signs in each group but when the evoked potentials were determined, a high proportion of all cats displayed AEP and VEP alterations. These data demonstrate the importance of these analyses in the detection of FIV-infected cats where neurological disease is not clinically evident. When results of VEP and AEP were compared before and after treatment, an improvement in the ZDV + Valp group and a worsening in the ZDV + rHuIFN-α group could be detected. The deterioration of neurological conditions observed in the ZDV + rHuIFNα group must be taken into account in the clinician’s consideration of IFN use for the treatment of FIV-infected cats, since IFN is not recommended when neurological disease is present. The neurological implications of IFN use in the treatment of lentiviral disease requires further research. In contrast, the administration of ZDV + Valp resulted in a marked improvement in neurological signs. This may reflect the depressor effect of valproic acid on the CNS.

Neither ZDV alone nor ZDV + 3TC showed changes in either neurological signs or in VEP and AEP. This may reflect the poor penetration of these antiviral drugs into the CNS [10]. The addition of TL3 to the ZDV + 3TC combination may be useful to prevent the entry of the virus into the CNS [4,5]. 

Throughout the one year of evaluation, very few therapeutic side effects were noted, most likely because the cats selected for study had near normal CD4+/CD8+ ratios at the beginning of the study. When cats are in poor health, they are more likely to suffer side effects of the therapy. However it is possible that the low incidence of side effects was related to strict dose control and the permanent follow-up of the patients. 

## 3. Experimental Section

### 3.1. Population under Study

Thirty-two cats, between 6–10 years, with naturally acquired FIV infection were enrolled in the study. Forty-six percent were female, 56% were male, 80% of mixed breed, 20% of several pure breeds and all of them were owned, indoor cats. Anti-FIV antibodies were detected using an available commercial diagnostic kit for FIV antibody and FeLV antigen (*SpeedDUO-Medicatec*^®^) while viral nucleic acids were detected by PCR [18]. 

At the beginning of the study, all animals were healthy, displaying only occasional and mild clinical signs including gingivitis, lymphadenopathy and hyperglobulinemia. The cats were evaluated every two months from two to five years before the study by physical examination and by usual laboratory tests (hematology, biochemistry, assessment of opportunistic infections, ultrasonography and X-Ray). Viral loads of all cats were quantified once a year, while the CD4+/CD8+ ratio was calculated every four months. Cats were considered to be in the late phase of the “asymptomatic” stage and the treatment was started when the CD4+/CD8+ ratio reached 0.9, as at this stage of infection, the viral load increased markedly and clinical signs of immunosuppression began to appear.

The following opportunistic agents were evaluated: *Mycobacterium bovis*, *Cryptoccocus neoformans*, *Mycoplasma haemofelis*, *Toxoplasma gondii*, *herpesvirus*, *Rhodoccocus equi* and feline leukemia virus (FeLV). The study groups were formed in a randomized fashion as follows: 2 cats for each group according to the animals that were brought at the hospital, beginning with the ZDV group and continuing as follows:

- ZDV group: eight cats received treatment with Zidovudine, 5 mg/kg/12 h, oral, during one year.
- ZDV + rHuIFN: eight cats treated with Zidovudine and rHuIFNα, 1 mU/Kg/ SC/ 24 h, 3 cycles every one week, one month apart.
- ZDV + Valp: eight cats treated with Zidovudine and valproic acid 15 mg/kg/24 h.
- ZDV + 3TC: eight cats treated with Zidovudine and 3TC 25 mg/kg/12 h/year.

The follow-up of the treated cats was performed with periodic physical examination and the usual laboratory tests during the following 12 months. In all groups, CD4+/CD8+ ratio and viral load were enumerated while VEP and AEP determinations were performed at the beginning of the study and after one year.

### 3.2. Disease Diagnosis

The initial FIV diagnosis was performed using a commercially available immunochromatographic test kit (*SpeedDUO-Medicatec*^®^), according to the manufacturer’s instructions. Once the cats were received into our study, FIV infection was confirmed by PCR developed in our institution previously, as a second diagnostic step [18,22]. 

### 3.3. Determination of the CD4^+^/CD8^+^ Ratio using Flow Cytometry

Blood samples were collected using an EDTA anticoagulant. The mononuclear layer was separated with Histopaque (Sigma Diagnostics) and marked with monoclonal antibody to the CD4 antigen (fluorescein isothiocyanate (FITC)-conjugated mouse anti-cat CD4-FITC, Abd Serotec) and CD8 antigen (phycoerithrin (PE)-conjugated mouse anti-feline CD8αβ-RPE). The samples were analyzed in a flow cytometer (Becton Dickinson FACScalibur, Becton Dickinson Biosciences) in the Cassará Laboratory (Buenos Aires, Argentina). The ratio was considered as having decreased when it reached < 0.9. The CD4+/CD8+ ratio was measured at the beginning and end of the study in the four groups.

### 3.4. Viral load determination (VL)

In order to evaluate VL, we used a previously described technique [22,24]. FIV RNA was detected and quantified by quantitative competitive PCR. Briefly, RNA was extracted from 140 µl of cell-free plasma with a commercial kit *QIAamp®Viral RNA* (*Qiagen*) under PCR-clean conditions, and pelleted. Products were amplified by quantitative competitive PCR with *SuperScript™ One-Step RT-PCR with Platinum Taq* (*Invitrogen*) for the FIV *gag* sequences in order to quantify the FIV genomes in plasma. The RNA competitor was produced *in vitro* by differential amplification using the wild-type gag sequence of 312 bp as a target. We obtained a 98 bp RNA competitor. The RNA transcripts were then purified from the DNA templates by digestion with RNAse-free DNAse, phenol-chloroform extraction, and alcohol precipitation; resuspended in nuclease-free water and quantified spectrophotometrically at 260 nm. The RT-cPCR was carried out by the addition of 5µl of plasma RNA to 5 µL of serial 10-fold dilutions (10^10^ to 10^7^) of RNA competitor and 40µl of a mixture containing 40 pmolof each FIV *gag* primers; sense 771 (AGAACCTGGTGATATACCAGAGAC) antisense 1081 (TTGGGTCAAGTGCTACATATT), 2 mM Mg Cl_2_, 100 UI of *Recombinant RNAsin Ribonuclease Inhibitor* (*Promega*) and 2 U of *Taq* DNA polymerase (*SuperScript™One-Step RT-PCR with Platinum Taq* (*Invitrogen*)). The analysis of PCR products was performed by agarose gel electrophoresis with *Image J 1.38x* software *(National Institute of Health*, *USA)*. Analytical sensitivity was estimated at approximately 10^6^ copies/mL. The sensitivity of this technique allowed us to distinguish the transition from the asymptomatic carrier stage to the AIDS stage and was measured at the beginning and at the end of the study in all groups. 

### 3.5. Visual Evoked Potentials (VEP) and Auditory Evoked Potentials (AEP)

Electrical potentials produced in response to auditory stimulation are called auditory evoked potentials (AEP). The principal means of assessing auditory function in animals is the brain stem auditory evoked response (AEP). Short duration auditory stimuli, such as clicks and responses are recorded by electrodes. AEP may be used to assess lesions in the brain stem. In FIV-infected cats, AEP present diminished waves and increased latencies and this indicates a central conduction defect. 

In contrast, the light stimulus produces cortical responses that are referred to as visual evoked potentials (VEP). The most common alteration in the VEP is a decrease of the P100 wave amplitude and a prolonged latency in cats naturally infected with FIV.

AEP and VEP were evaluated by the Service of Electrophysiology at the Veterinary Medicine Teaching Hospital of the University of Buenos Aires with an ATI, Nautilus equipment. Both determinations are used routinely by this service and have been standardized since 1999 [21,25]. VEP and AEP were measured at the beginning and at the end of the study in all groups. 

The VEP, using sub-dermal needle electrodes, were recorded between Oz (midline of the nuchal crest, positive electrode) and Fpz (midline, just caudal to the eyes, negative electrode), with ground at Cz (vertex). The stimulus is provided by stroboscopic flashes. The registers were recorded in the dark with dark adaptation (e.g., 15 minutes). No sedative drugs or mydriatics were used. These potentials are important in distinguishing between visual problems produced by eye disorders due to lesions in the visual pathway distal to the eye. Normal values: latency to 100 milliseconds (msec.). 

The AEP were obtained using sub-dermal needle electrodes placed over the vertex (negative) and just rostral to the base of each ear. Each electrode was used as a reference for an ispilateral recording, while the other was grounded. These electrical potentials are produced in response to auditory stimulation. All recordings were obtained without sedation. Short duration auditory stimuli (80 dB), such as clicks, and the responses were recorded with sub-dermal needle electrodes. The value of the normal central time of conduction was until 4–8 msec.

### 3.6. Statistical Analysis

Results were expressed as median and range, and p < 0.05 was considered significant. Statistical analyses were performed using non-parametric ANOVA (Kruskal Wallis) followed by Dunn's test. The comparative analysis intra group (pre-treatment *vs.* one year) was carried out with Wilcoxon's test.

### 3.7. Ethical Approval

The study was approved by the Institutional Animal Care and Use Committee of the Faculty of Veterinary Sciences of the University of Buenos Aires and the Secretary of Science and Techniques of the aforementioned University (UBACyT) fulfilling the national laws on experiments with animals. The cat’s owners gave their signed consent for the participation of their animals in this study.

## 4. Conclusions

Both ZDV alone and the combined therapy ZDV + 3TC were well tolerated in cats with minimal side effects. Although both therapies were effective at improving the CD4+/CD8+ ratio and inducing a decrease in viral load in comparison to the other treatment combinations, cats treated with ZDV + 3TC showed a greater decrease in viral load than those treated with ZDV alone. Therefore, the combination ZDV + 3TC could be more effective than the use of ZDV alone. The results from the evoked potential analyses revealed a high incidence of neurological alterations, without evident clinical signs. A worsening of neurological signs was observed in the ZDV + rHuIFNα treated group, whereas in the ZDV + Valp treated cats there were improvements in neurological signs. Taken together, the results presented here suggest that the combination of ZDV + 3TC is an effective treatment for FIV-infected cats in the late phase of the “asymptomatic” stage of infection and may prevent the manifestation of the feline AIDS stage. 
